# First Report of a *bla*_OXA-484_-Harbouring *Escherichia coli* ST167 Isolated from the Urine Sample of a Dog of Italian Origin

**DOI:** 10.3390/antibiotics15070652

**Published:** 2026-06-30

**Authors:** Michael Biggel, Magdalena Nüesch-Inderbinen, Sarah Schmitt, Marianne Schneeberger, Natalie Hofer, Jule Anna Horlbog, Roger Stephan

**Affiliations:** 1 Institute for Food Safety and Hygiene, Vetsuisse Faculty, University of Zurich, 8057 Zurich, Switzerland; michael.biggel@uzh.ch (M.B.); magdalena.nueesch-inderbinen@uzh.ch (M.N.-I.); jhorlbog@fsafety.uzh.ch (J.A.H.); 2 Section of Veterinary Bacteriology, Institute for Food Safety and Hygiene, Vetsuisse Faculty, University of Zurich, 8057 Zurich, Switzerland; sarah.schmitt@vetbakt.uzh.ch (S.S.); marianne.schneeberger@vetbakt.uzh.ch (M.S.); 3 Clinic for Small Animal Internal Medicine, Vetsuisse Faculty, University of Zurich, 8057 Zurich, Switzerland; natalie.hofer@uzh.ch

**Keywords:** carbapenemase, OXA-484, *E. coli* ST167, canine infection, dog, transmission

## Abstract

Antimicrobial resistance (AMR) is a global threat to both human and animal health. Carbapenems are last-resort antimicrobials used to treat severe infections with multidrug-resistant Gram-negative nosocomial pathogens in humans. Therefore, the dissemination of carbapenemase-producing Enterobacterales (CPE) has emerged as a major concern worldwide. Although carbapenems are not routinely used in veterinary medicine, CPE, including OXA-48-like-producing *Escherichia coli*, are increasingly being reported in companion animals. We document the first report of *E. coli*-harbouring *bla*_OXA-484_ isolated from a urine sample from a dog with a history of chronic thoracolumbar myelopathy. Using a combined Oxford Nanopore (ONT) long-reads and Illumina short-reads sequencing approach, the isolate was characterized and an IncF plasmid containing *bla*_OXA-484_ was reconstructed. The isolate belonged to sequence type (ST)167, which is an emerging high-risk clone frequently reported among human clinical isolates. The *bla*_OXA-484_ gene was harboured in a composite transposon bracketed by IS26 identical to that of *bla*_OXA-484_ carried on an IncX plasmid pOXA-484-JS316 from a human clinical *E. coli* ST410 from Germany. The isolation of the epidemic clone ST167 harbouring *bla*_OXA-484_ from a canine infection raises the hypothesis of a transmission event between humans and companion animals.

## 1. Introduction

Antimicrobial resistance is a threat to both human and animal health. Carbapenems are last-resort antimicrobials used to treat severe infections with multidrug-resistant (MDR) Gram-negative nosocomial pathogens in humans [1]. The dissemination of carbapenemase-.producing Enterobacterales (CPE) is a major concern worldwide [2]. The production of OXA-48–like enzymes is one of the most prevalent mechanisms of carbapenem resistance [2]. So far, more than 55 variants of OXA-48 have been identified. OXA-484 differs from OXA-48 by 5 amino acid substitutions (Thr104Ala, Asn110Asp, Glu168Gln, Ser171Ala, and Arg214Gly), and from the closely related OXA-181 by Arg214Gly [3]. OXA-484-producing *E. coli* belonging to sequence type (ST) 410 or ST1722 emerged in clinical isolates in France from 2021 to 2023 [4]. Although carbapenems are not routinely used in veterinary medicine, Enterobacterales expressing OXA-48 have been reported in companion animals in Germany and France [5]. However, the OXA-484 variant has so far not been recorded in clinical veterinary isolates.

Here, we describe for the first time the isolation of a *bla*_OXA-484_-harbouring *E. coli* isolated from a urine sample from a dog and present the complete genome sequence of this multidrug-resistant isolate.

## 2. Results and Discussion

*E. coli* 51243816-SK1A was cultured from a urine sample obtained from a dog that was admitted to the Department of Small Animals of the University of Zurich.

Strain identification and routine antimicrobial susceptibility profiling using the VITEK^®^ 2 system identified multidrug-resistant *E. coli* with the following minimal inhibitory concentrations (MICs) (µg/mL): ampicillin ≥ 32; amoxicillin/clavulanic acid ≥ 32/16; cefalexin ≥ 64; cefpodoxime ≥ 8; cefovecin ≥ 8; ceftiofur ≥ 8; imipenem 2; gentamicin ≤ 1; amikacin ≤ 2; enrofloxacin ≥ 4; marbofloxacin ≥ 4; pradofloxacin ≥ 4; doxycycline ≥ 16; tetracycline ≥ 16; chloramphenicol 16; nitrofurantoin 32. *E. coli* 51243816-SK1A was therefore phenotypically resistant to ampicillin, amoxicillin/clavulanic acid, cefalexin, cefpodoxime, cefovecin, ceftiofur, enrofloxacin, marbofloxacin, pradofloxacin, doxycycline and tetracycline.

Due to the increased MIC for imipenem, the NG-Test Carba 5 (NG-Biotech Laboratoires) was performed, with a positive result for an OXA-48-like carbapenemase.

Whole-genome sequencing revealed that *E. coli* 51243816-SK1A belonged to ST167, an emerging high-risk clone in human clinical isolates, and harboured three plasmids (Table 1). The largest plasmid, p51243816-SK1A-A (113.3 kb), belonged to the IncF family of replicons and carried multiple antimicrobial resistance genes, including *bla*_OXA-484_, conferring resistance to carbapenems, and the ciprofloxacin resistance gene *qnrS1* (Table 1). A second plasmid, p51243816-SK1A-B (67.4 kb), was classified as IncI(Gamma) and encoded the AmpC-type cephalosporinase *bla*_CMY-145_. The third plasmid, p51243816-SK1A-C (61.7 kb), belonged to IncI2(Delta) and did not contain any known antibiotic-resistance determinants. All three plasmids were predicted to be conjugative by the Mobtyper tool. No important virulence genes were found on the plasmids.

*E. coli* 51243816-SK1A also carried chromosomal point mutations in *gyrA* (S83L, D87N), *parC* (S80I), and *parE* (S458A), which are associated with resistance to nalidixic acid and ciprofloxacin.

**Figure 1:**
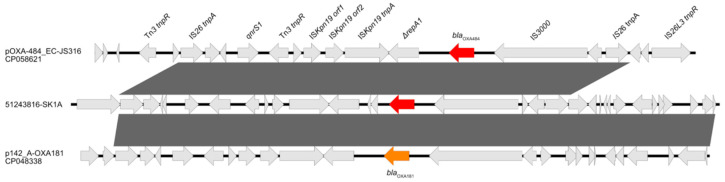
Genetic context of *bla*_OXA-484_ in plasmid p51243816-SK1A-A compared to the genetic context of *bla*_OXA-484_ in pOXA-484_EC-JS316 (CP058621) carried by a human clinical *E. coli* ST410 [6], and of *bla*_OXA-181_ in p142_A-OXA181 (CP048338) purified from an environmental *E. coli* ST648 [7]. Grey shades between sequences indicate regions with >99% sequence identity. The figure was generated using EasyFig v2.1 (https://mjsull.github.io/Easyfig/files.html, accessed on 16 January 2026).

Plasmid p51243816-SK1A-A harboured *bla*_OXA-484_ and *qnrS1* in a composite transposon bracketed by IS26 (Figure 1).

This genetic context corresponds with >99% sequence identity to that of *bla*_OXA-484_ on the 51.5 kb IncX plasmid pOXA-484-JS316 carried by an *E. coli* ST410 isolated in 2019 from a German patient with history of travel to India [6] (Figure 1). Furthermore, the region encompassing *bla*_OXA-484_ is identical to that of *bla*_OXA-181_ encoded on the 155 kb IncFIB mosaic plasmid p142_A-OXA-181 carried by *E. coli* ST648 isolated from river water in Switzerland [7] (GenBank accession no. CP48338.1) (Figure 1), except for a point mutation within the *bla*_OXA_ gene itself. These similarities suggest that *bla*_OXA-484_ is likely the result of a mutation in *bla*_OXA-181_ and is associated with potential mobilization among IncX3 and IncF-type plasmids. Most previously reported OXA-484 producers belong to *E. coli* ST410 and *E. coli* ST1722 associated with human infections in France, Germany, and the UK [3,4,6]. Recently, *E. coli* ST167 harbouring *bla*_OXA-484_, *bla*_CMY-146_, and *qnrS1* was isolated from a patient in the United Arab Emirates [8]. This study is based on a unique *E. coli* isolate. Notwithstanding this limitation, our findings are of clinical relevance. The emergence of this successful epidemic clone ST167 harbouring *bla*_OXA-484_ among canine *E. coli* isolates in Switzerland raises the hypothesis of a human to animal transmission event. However, further information such as the dog’s history of hospitalization, antibiotic exposure, contact with humans, travel, or exposure to environmental sources would be needed to corroborate this interpretation. The detection of *E. coli* ST167 harbouring *bla*_OXA-484_ isolated from a companion animal highlights the importance of veterinary diagnostic stewardship and the need for infection prevention in veterinary clinics and pet owner education in order to mitigate the spread of AMR and protect human and animal health.

## 3. Materials and Methods

### 3.1. Bacterial Strain and Antimicrobial Susceptibility Testing

*Escherichia coli* 51243816-SK1A was isolated from a urine sample from an eleven-year-old spayed female French Bulldog, originally from Italy (Cupra Marittima), that was presented to the Department of Small Animals of the University of Zurich with a history of chronic thoracolumbar myelopathy with acute deterioration of neurological signs and urinary incontinence.

Antimicrobial susceptibility testing was conducted with a VITEK^®^ 2 (bioMérieux, Marcy l’Etoile, France) using the AST-GN97 card according to manufacturer’s instruction. The advanced expert system (AES) of the VITEK^®^ 2 provided minimal inhibitory concentration (MIC) and susceptibility interpretations. Interpretation of results referred to the guidelines and animal species-specific breakpoint interpretations of the Clinical and Laboratory Standards Institute (VET01S, CLSI M100) (https://clsi.org/shop/standards/vet01s/ (accessed on 6 December 2025)). If no dog-specific clinical breakpoints were available, cat-specific or human-specific breakpoints were used for interpretation. The presence of a carbapenemase was confirmed using the NG-Test Carba 5 (NG-Biotech Laboratoires, Guipry, France).

### 3.2. Whole-Genome Sequencing and Analysis of Whole-Genome Sequencing Data

The whole-genome sequence of *E. coli* 51243816-SK1A was determined using short-read (Illumina MiniSeq, Illumina, San Diego, CA, USA) and long-read sequencing (MinION, Oxford Nanopore Technologies, Oxford, UK). The isolate was grown on sheep blood agar at 37 °C overnight prior to DNA isolation using the MagPurix Bacterial DNA Extraction Kit (Zinexts, Châtel-St-Denis, Switzerland). Illumina libraries were prepared using a Nextera DNA Flex Sample Preparation Kit (Illumina, San Diego, CA, USA), and sequenced on an Illumina MiniSeq (Illumina). For long-read sequencing, libraries were prepared using the SQK-RBK114.96 Rapid Barcoding Kit (Oxford Nanopore, Oxford, UK) according to the “Nanopore-only Microbial Isolate Sequencing Solution” protocol. Libraries were sequenced on a P2Solo device using R10.4.1 flow cells. Illumina read adapters and low-quality bases were trimmed with fastp 0.22.0 (https://github.com/OpenGene/fastp). Dorado 1.2.0 (https://github.com/nanoporetech/dorado) with the sup@v5.2 (dna_r10.4.1_e8.2_400bps_sup@v5.2.0) model was used for simplex basecalling, demultiplexing, and adapter and barcode trimming of Nanopore data. Nanopore reads were filtered to a minimum read length of 1000 bp and quality controlled using nanoq 0.10.0 (https://github.com/esteinig/nanoq). Hybrid assemblies were generated using Unicycler v0.5.0 (https://github.com/rrwick/Unicycler) with default settings. Sequence types were identified with mlst 2.22.0 (https://github.com/tseemann/mlst), which relies on PubMLST (https://pubmlst.org). Antimicrobial resistance genes were identified using AMRFinderPlus 4.2.5 with database 2025-12-03.1 (https://github.com/ncbi/amr/releases/tag/amrfinder_v4.2.5). Plasmid replicon types were identified with ABRicate v1.0.0 (https://github.com/tseemann/abricate), in conjunction with the PlasmidFinder database (minimum sequence coverage/identity 70%/90%) (https://github.com/genomicepidemiology/plasmidfinder). Mobtyper version 3.1.9 (https://github.com/phac-nml/mob-suite) was used to predict plasmid transferability. Plasmids were screened for the presence of virulence factors using the Virulence Factor Database VFDB (https://www.mgc.ac.cn/VFs/). All URLs were accessed on 9 January 2025.

## Figures and Tables

**Table 1:** Features of the three plasmids harboured by *Escherichia coli* 51243816-SK1A.

Plasmid	Size (bp)	Replicon Type [pMLST] ^a^	Relaxes Type	*ori*T	Antibiotic Resistance Determinants ^b^	ENA Accession Number
p51243816-SK1A-A	113,340	IncFIA, IncFIB, IncFII, Col156, ColKP3 [F1:A1:B66] *	MOBF, MOBP	MOBF	*bla*_OXA-484,_*dfrA14*, *erm(B), mph(A)*, *qnrS1*, *tetB*	OZ409597
p51243816-SK1A-B	67,395	IncI(Gamma) [–]	MOBP	MOBP	*bla*_CMY-145_	OZ409598
p51243816-SK1A-C	61,681	IncI2(Delta) [–]	MOBP		–	OZ409599

^a^ pMLST: Allele numbers and sequence types assigned by plasmid pMLST web tool at https://cge.food.dtu.dk/services/pMLST (accessed on 9 January 2026). ^b^ Carbapenem resistance gene: *bla*_OXA-484_; cephalosporin resistance gene: *bla*_CMY-145_; macrolide resistance genes: *erm(B)*, *mph(A)*; trimethoprim resistance gene: *dfrA14*; quinolone resistance gene: *qnrS1*; tetracycline resistance gene: *tetB.* * Partial match with FIB 66 in the pMLST database. –, not identified.

## Data Availability

The data for this study have been deposited in the European Nucleotide Archive (ENA) at EMBL-EBI under accession number PRJEB107994 (https://www.ebi.ac.uk/ena/browser/view/PRJEB107994 (accessed on 10 February 2026)), and assembly accession number GCA_979243125.

## Abbreviations

The following abbreviations are used in this manuscript:

AMR	Antimicrobial resistance
Ala	Alanine
Arg	Arginine
Asn	Asparagine
Asp	Aspartic acid
CLSI	Clinical and Laboratory Standards Institute
CPE	Carbapenemase-producing Enterobacterales
Gln	Glutamine
Glu	Glutamic acid
Gly	Glycine
ONT	Oxford Nanopore
Ser	Serine
ST	Sequence type
Thr	Threonine
WHO	World Health Organization
